# Structure-Guided Engineering of Molinate Hydrolase for the Degradation of Thiocarbamate Pesticides

**DOI:** 10.1371/journal.pone.0123430

**Published:** 2015-04-23

**Authors:** José P. Leite, Márcia Duarte, Ana M. Paiva, Frederico Ferreira-da-Silva, Pedro M. Matias, Olga C. Nunes, Luís Gales

**Affiliations:** 1 Instituto de Investigação e Inovação em Saúde, Universidade do Porto, Porto, Portugal; 2 IBMC—Instituto de Biologia Molecular e Celular, Universidade do Porto, Porto, Portugal; 3 LEPABE—Laboratory for Process Engineering, Environmental, Biotechnology and Energy, Faculdade de Engenharia da Universidade do Porto, Porto, Portugal; 4 Instituto de Tecnologia Química e Biológica António Xavier, Universidade Nova de Lisboa, and IBET, Apartado 12, 2781–901 Oeiras, Portugal; 5 ICBAS—Instituto de Ciências Biomédicas Abel Salazar, Universidade do Porto, Porto, Portugal; Griffith University, AUSTRALIA

## Abstract

Molinate is a recalcitrant thiocarbamate used to control grass weeds in rice fields. The recently described molinate hydrolase, from *Gulosibacter molinativorax* ON4^T^, plays a key role in the only known molinate degradation pathway ending in the formation of innocuous compounds. Here we report the crystal structure of recombinant molinate hydrolase at 2.27 Å. The structure reveals a homotetramer with a single mononuclear metal-dependent active site per monomer. The active site architecture shows similarities with other amidohydrolases and enables us to propose a general acid-base catalysis mechanism for molinate hydrolysis. Molinate hydrolase is unable to degrade bulkier thiocarbamate pesticides such as thiobencarb which is used mostly in rice crops. Using a structural-based approach, we were able to generate a mutant (Arg187Ala) that efficiently degrades thiobencarb. The engineered enzyme is suitable for the development of a broader thiocarbamate bioremediation system.

## Introduction

Molinate (S-ethyl azepane-1-carbothioate) is one of the most intractable thiocarbamates [1] and is extensively used worldwide to control grass weeds in rice crops. Most of the known pathways for molinate degradation lead to the formation of partially oxidized metabolites, which are more toxic and persistent than the parent compound [2]. So far, there is only one described microbial system able to degrade molinate to innocuous compounds: a five strong bacteria consortium from which *Gulosibacter molinativorax* ON4^T^ is responsible for the initial breakdown of the herbicide by cleaving its thioesther bond, releasing ethanethiol and azepane-1-carboxilate (ACA) [3, 4]. This reaction is catalyzed by molinate hydrolase (MolA) [5]. For molinate concentrations over 2 mM and in the absence of other members of the bacterial consortium, *G*. *molinativorax* ON4^T^ growth is hindered, due to the accumulation of sulphur compounds, namely ethanethiol [3, 4]. By contrast, in the presence of other consortium members able to degrade the sulphur compounds, mineralization of the herbicide occurs even when the initial molinate concentration is close to its solubility (4 mM). Due to its role in the aforementioned molinate degradation pathway, a bioremediation tool with molinate hydrolase presents great value for environmental decontamination.

**Fig 1:**
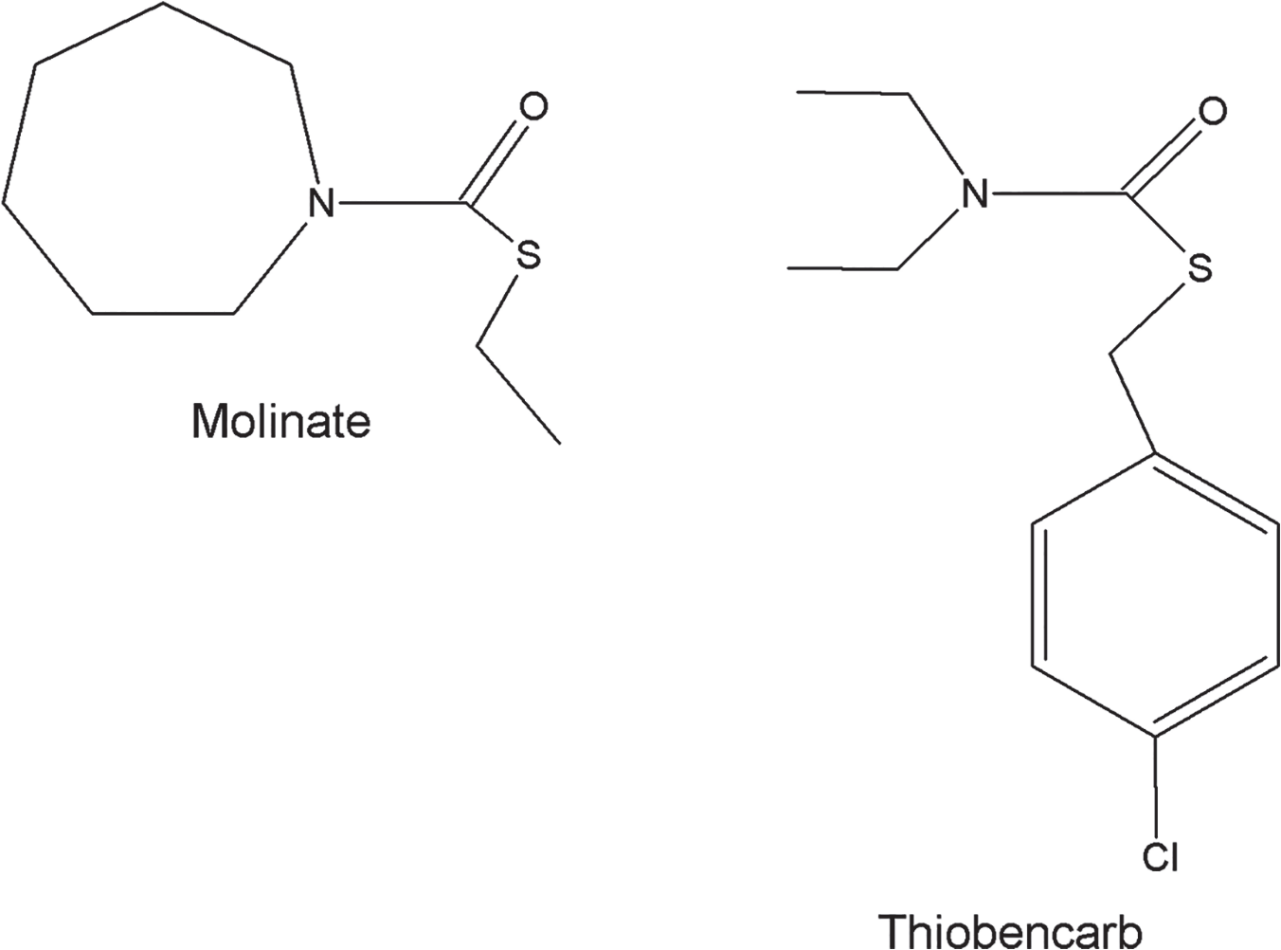
Two thiocarbamate herbicides commonly applied to rice crops.

Molinate hydrolase is a recently characterized metal-dependent enzyme [5]. Following successful identification and cloning of its encoding gene, recombinant molinate hydrolase was found to be cobalt-dependent, with zinc and manganese also able to confer activity to the enzyme [5]. Biochemical studies revealed that recombinant molinate hydrolase presents maximum activity at pH 7.5 and 30°C, showing very similar kinetic properties to native molinate hydrolase [5]. One of the most critical aspects of an enzyme is its substrate specificity. Alongside with molinate, other thiocarbamates are also used as pesticides. Similarly to molinate, thiobencarb is applied to rice crops (Fig 1) [2]. Molinate hydrolase proved to be unable to degrade other thiocarbamates such as thiobencarb, regardless of protein and/or substrate concentration [5], probably due to a steric effect. Therefore, the determination of the enzyme three-dimensional structure is of key importance for an eventual modulation of the enzyme specificity by site-directed mutagenesis.

Herein we present the crystal structure of molinate hydrolase. This allowed us to theorize a mechanism for molinate binding and consequent hydrolysis at the thioester bond, which constitutes a rare catalytic mechanism. We also demonstrate that molinate hydrolase is inhibited by ethanethiol, explaining the inhibition of *Gulosibacter molinativorax* ON4^T^ growth when this molinate hydrolysis product accumulates.

The disclosure of the crystal structure of the enzyme was crucial for the generation of single mutants that catalyse the degradation of other thiocarbamate pesticides, namely thiobencarb.

## Results and Discussion

### The overall crystal structure of molinate hydrolase

As molinate hydrolase shares similarity with other metal-dependent hydrolases, the metal dependence of this enzyme was shown in a previous report [5], where it was determined a metal ion / monomer ratio of 1.1 ± 0.2 by both ICP-AES and ICP-MS. Incubation with chelating agents rendered an inactive form, which could be reactivated through the addition of divalent metal ions [5]. However, despite of several attempts, crystallization trials yielded crystals of the metal-depleted amidohydrolase. These results suggest that molinate hydrolase loses the catalytic ion during crystallization trials, a event that was reported before for other amidohydrolases [6]. Thus, we produced the SeMet-mutant and determined the three-dimensional structure of the enzyme by the single wavelength anomalous diffraction (SAD) technique using the anomalous signal from the selenium atoms. Later on we solved a crystal structure partially loaded with one of the potential catalytic metal ions, Zn^2+^, by co-crystallizing the recombinant enzyme with ZnCl_2_. The position of the metal ions was determined from single wavelength anomalous diffraction data collection at the Zn absorption K-edge. The anomalous signal was modest but the top positive peaks in the anomalous difference Fourier map were located in equivalent positions in each monomer and were consistent with metal coordination geometry and with the position of the metal ions in structure-related amidohydrolases. Nevertheless we performed site-directed mutagenesis of the metal-coordinating residues. Inactive mutants were obtained, confirming the structure of the active site. The Zn ions occupation was set to 0.4 and the overall structure was similar to the one of the metal–free enzyme (root-mean-squared-distance r.m.s.d. of the superimposed structures is 0.22 Å). The active site structure was also not perturbed by the depletion of the catalytic metal ion.

Good diffracting crystals of recombinant molinate hydrolase were obtained in two distinct crystal systems, orthorhombic and monoclinic, with 4 and 8 monomers in the asymmetric unit, respectively. No significant differences were observed between the molinate hydrolase three-dimensional structures in the two crystal systems (r.m.s.d. of the superimposed structures is 0.17 Å).

**Fig 2:**
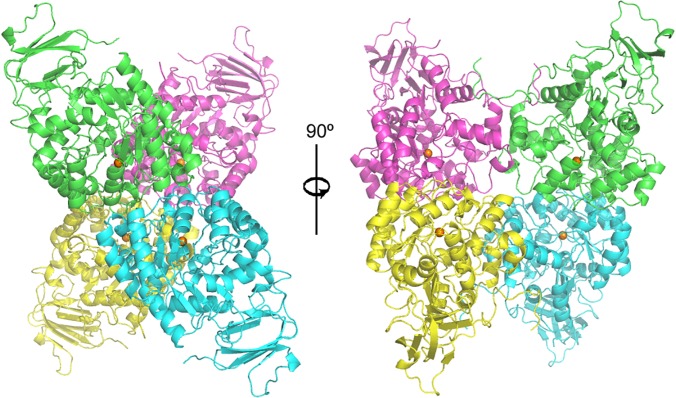
Overall structure of recombinant molinate hydrolase homotetramer. The monomers are coloured individually and the zinc cofactors are represented by orange spheres.

**Fig 3:**
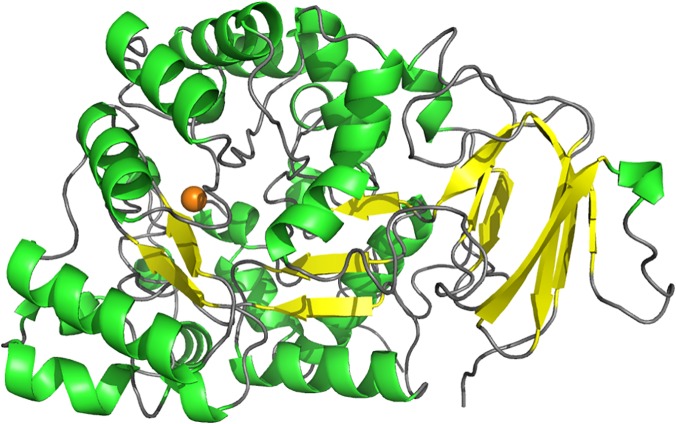
Structure of recombinant molinate hydrolase monomer highlighting the protein secondary structure. The zinc cofactor is represented by the orange sphere and the side chains of the active site residues are shown as sticks. α-helices are predominant, although a β-sandwich domain and two short β-sheets are also present.

The structure of molinate hydrolase consists of a homotetramer (Fig 2) composed of two symmetry-related dimers with a 67° tilt angle between them. The two dimers are assembled into the active enzyme by interactions involving the C-termini and α-helices. Within the dimer, the monomers are tightly bound, mostly through extensive interactions involving the residues in the loops connecting the α-helices. Each monomer binds a single divalent metal and is formed mostly by α-helixes (Fig 3). They contain also a β-sandwich domain characterized by a four-stranded and a three-stranded antiparallel opposing β-sheets, and two short three-stranded parallel β-sheets.

**Fig 4:**
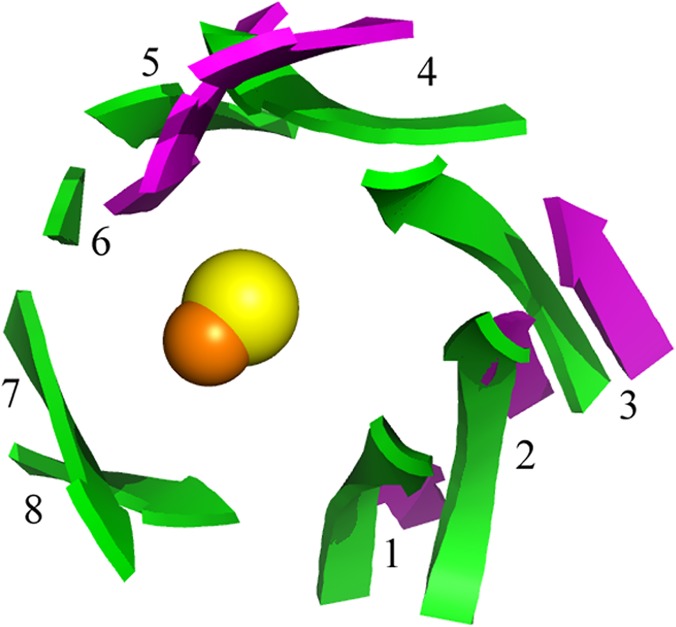
β-sheet arrangement around the catalytic site. Overlay of the β-strands around the catalytic metal of molinate hydrolase (pink) and N-acetyl glucosamine-6-phosphate deacetylase (green; PDB entry: 1o126), an enzyme that shows the classic β-barrel core (green) of the amidohydrolase superfamily; the usual β-strand numbering of the β-barrel is presented. The C_α_ of the metal coordinating residues were used for structure superposition. Catalytic metals are shown as spheres (zinc orange; iron yellow).

The most notable feature about the structure of molinate hydrolase is the absence of the 8-stranded β-barrel characteristic of the amidohydrolase superfamily. Despite the positional similarity of the active site when compared to other amidohydrolases, as will be detailed later, only six β-strands are conserved, forming two β-sheets. Those β-sheets are arranged to resemble an incomplete β-barrel, as can be observed after superposition with other amidohydrolases (Fig 4), such as *N*-acetyl glucosamine-6-phosphate deacetylase from *Thermotoga maritima* (PDB entry: 1o12; [6]). Both enzymes share high similarities in the active site geometry as will be discussed below.

### The structure of the active site

**Fig 5:**
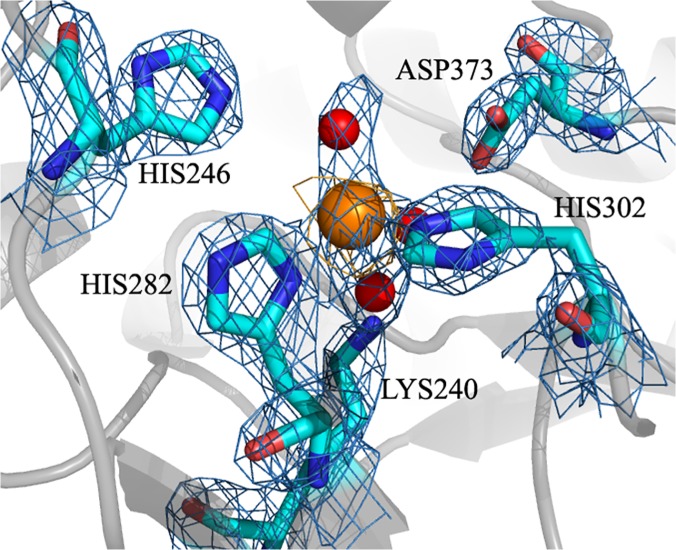
The active site of recombinant molinate hydrolase (detailed view; background in cartoon representation; highlighted residues in stick representation, with carbon backbone in cyan, oxygen red and nitrogen dark blue; the Zn cofactor is shown as an orange sphere and water molecules as red spheres). The anomalous difference map computed from the diffraction data measured at the Zinc absorption edge K and contoured at 4σis shown in orange mesh, and the 2Fo-Fc electron density map at 2σis drawn as a blue mesh around the side chains of the active site residues.

The catalytic metal is coordinated by His282 and His302 (residues are numbered according to the recombinant protein sequence, including a 32 amino acid purification tag), which are hydrogen-bonded to the main chain carbonyl groups of Asp245 and Thr283, respectively, as well as three ordered water molecules. The water molecules are in turn hydrogen-bound to Lys240, His246 and Asp373 (Fig 5). His282 and His302 are located in the loops after strands β5 and β6, respectively, while Lys240 is in strand β4 and His246 and Asp373 belong to loops that follow the β-sheet assembly.

**Fig 6:**
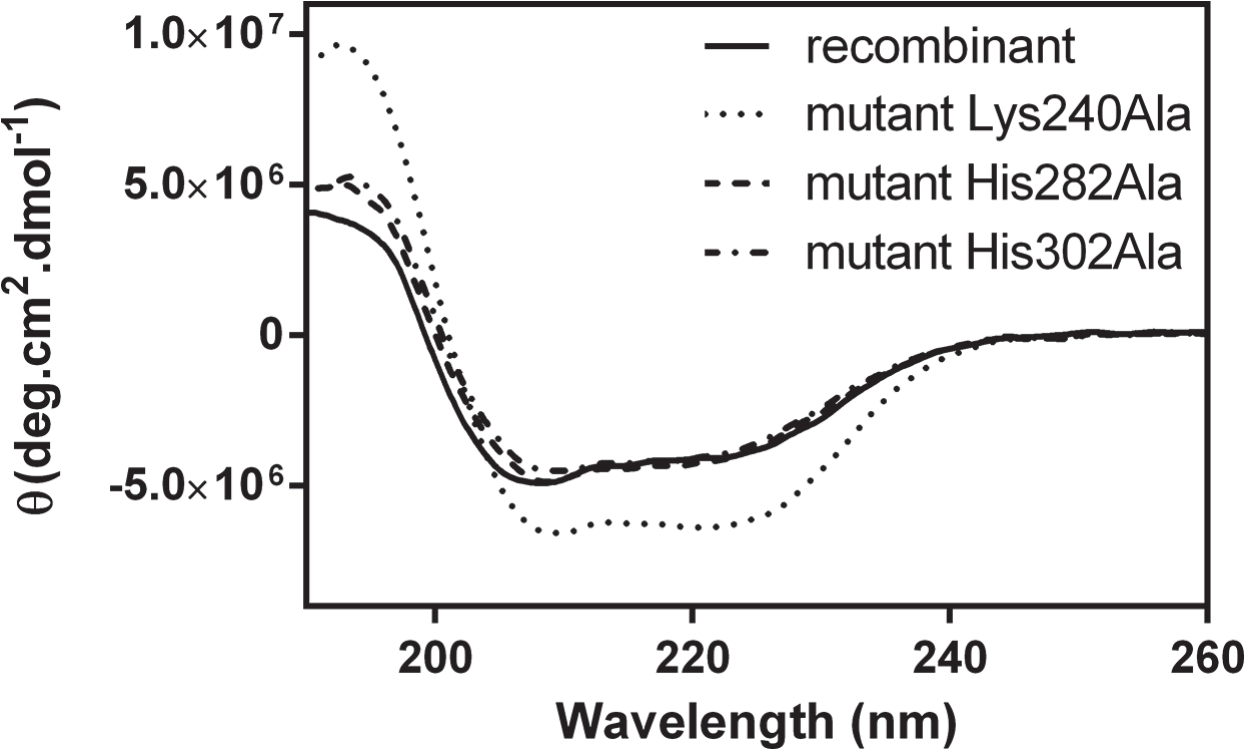
Circular dichroism spectra of the recombinant molinate hydrolase and of the mutants Lys240Ala, His282Ala and His302Ala.

Mutagenesis confirmed that each of the metal coordinating residues, His282 and His302, as well as Lys240, which is hydrogen-bonded to a coordinating water molecule, play a role in the enzyme activity. Single point mutations to alanine of the three residues yielded inactive forms of the enzyme, supporting a catalytic role for the zinc ions found in the crystal structure. The circular dichroism spectra of the mutants were undistinguishable from the spectrum of the recombinant enzyme, confirming that the mutants were properly folded (Fig 6).

Molinate hydrolase belongs to the amidohydrolase superfamily [5] and displays >40% amino acid sequence homology with the herbicide degrading phenylurea hydrolases PhuA and PhuB [7]. His_a_-X-His_b_-X_83-140_-Lys-X_25-70_-His_c_-X_18-25_-His_d_-X_52-143_-Asp is a common metal binding motif of amidohydrolases [6, 8, 9]. It comprises two metal binding sites, the so-called M_α_ site coordinated by the two imidazole side chains of His_a_-X-His_b_ and the M_β_ site ligated by histidines His_c_-X_18-25_-His_d_; additionally, there is a highly conserved aspartate that either coordinates or is hydrogen-bonded to a hydrolytic water ligated to metal M_β_. Within this enzyme superfamily there are, however, examples of mononuclear and binuclear metal centres [6]. The binuclear metal centres are characterized by the simultaneous interaction of a carboxylated lysine or a glutamate with both metal ions, which are usually 3.6 Å apart.

**Fig 7:**
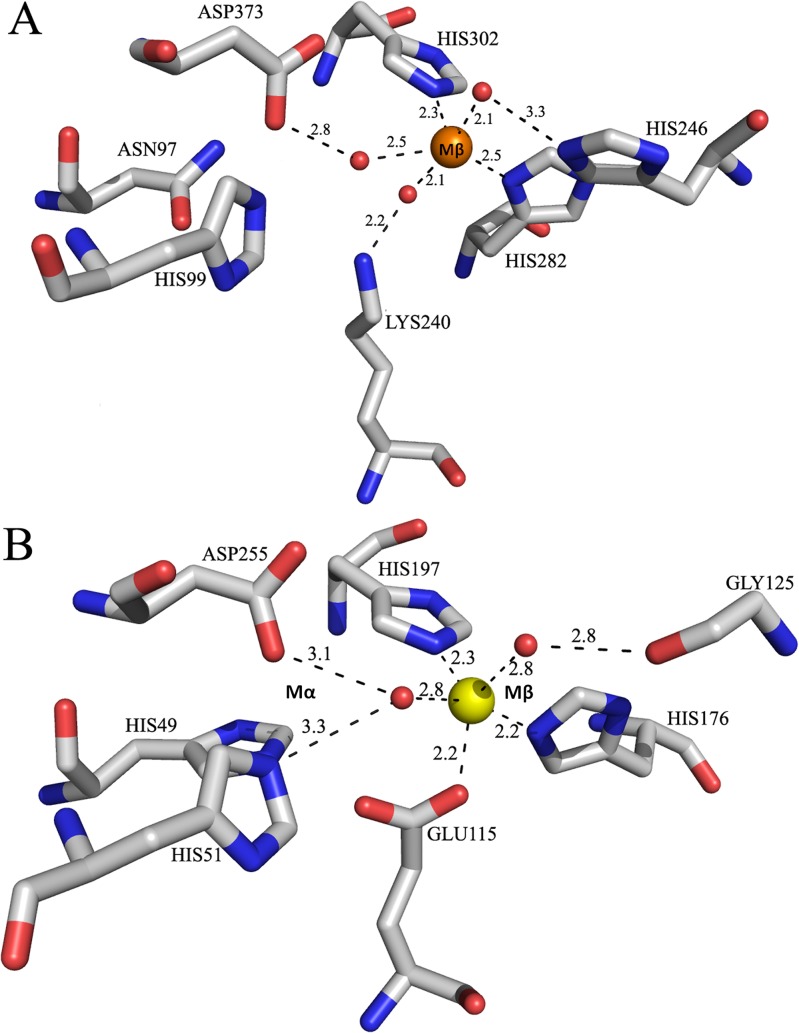
Active site homology. Active site structure homology between (A) recombinant molinate hydrolase and (B) subtype IV amidohydrolase *N*-acetyl glucosamine-6-phosphate deacetylase from *T*. *maritima* (PDB entry: 1o12; 6). Water molecules are represented as red spheres, zinc as an orange sphere and iron as a yellow sphere.

The molinate hydrolase structure revealed that the aforementioned lysine residue is definitely not carboxilated and that one of the histidines of the M_α_ site is substituted by an asparagine, as previously reported [5]. These observations, together with the lack of a positive electron density at the M_α_ site in the anomalous difference map calculated from diffraction data measured at the zinc absorption edge K, led us to conclude that molinate hydrolase has one catalytic metal ion per monomer located at the M_β_ site. As seen in Fig 7A, the divalent metal is pentacoordinated by His282, His302 and three water molecules. The water molecules are stabilized by Lys240, His246 and Asp373, respectively. This active site geometry is highly similar to that in *N*-acetyl glucosamine-6-phosphate deacetylase (AGD) from *T*. *maritima* (PDB file entry: 1o12; [6]), which has a single iron ion coordinated by two histidines (His176 and His197), a glutamate residue (Glu115) and two water molecules (Fig 7B). Glu115 appears to be positioned as a potential bridging residue between two catalytic metal ions and, in fact, in the AGD homologue from *Bacillus subtilis* (PDB file entry: 1un7[10]) there is a second metal ion at the M_α_ site.

### The catalytic mechanism

**Fig 8:**
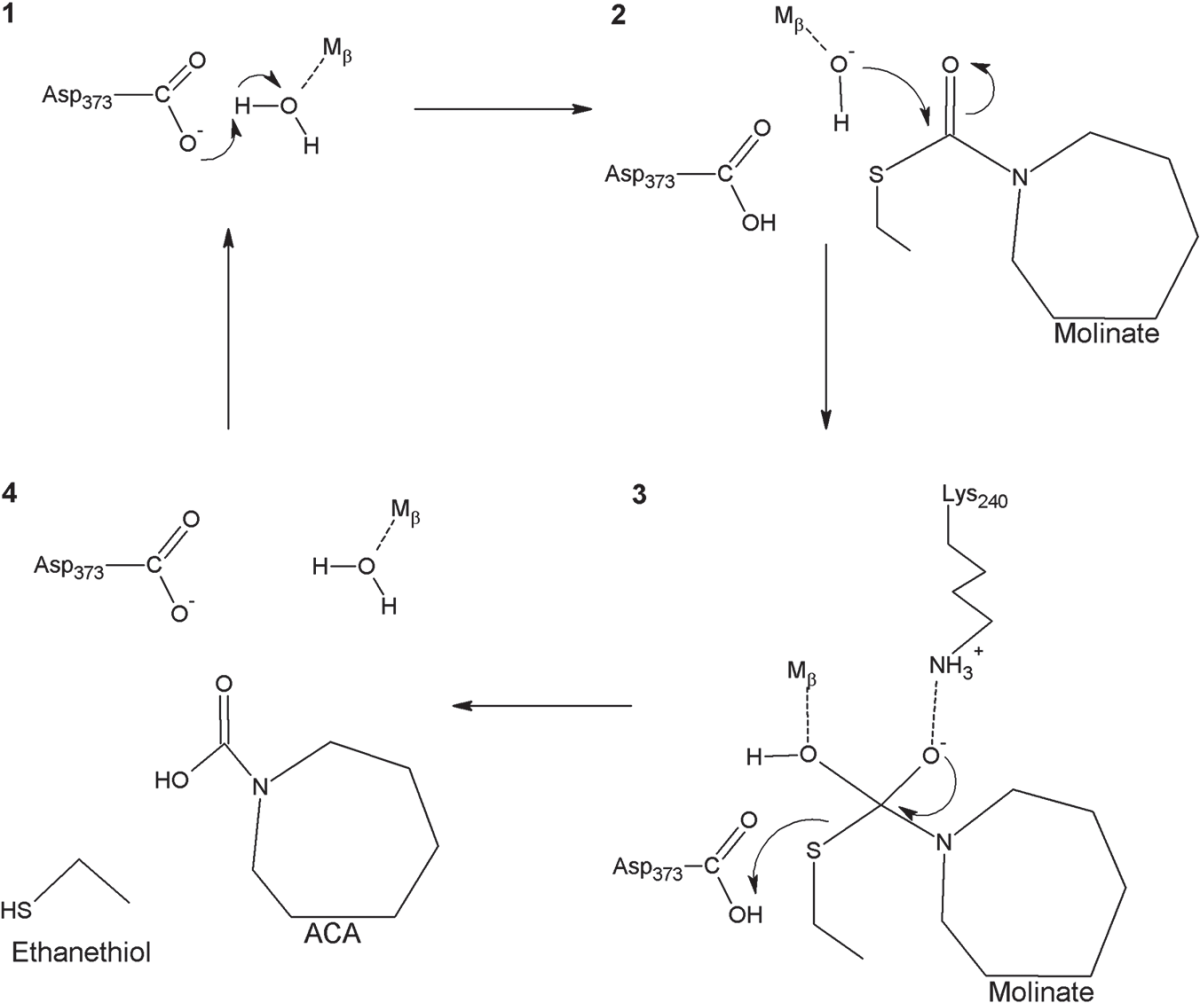
Proposed catalytic mechanism for molinate hydrolase based on previous models [6, 7].

Molinate hydrolase shares strong similarities with phenylurea hydrolases regarding the active site geometry and the pH dependence of kinetic parameters, i.e., a maximum enzyme activity between pH 6.5 and 8.5 [5, 7]. Thus, it is rather plausible that this enzyme follows the consensual catalytic mechanism characterized by a nucleophilic hydroxide, a metal-bound water molecule and an attack to the carbonyl group while a catalytic acid residue protonates the amide nitrogen—in our rare case the thiol sulphur atom [8, 9, 11] (Fig 8). The protonated intermediate is then decomposed by the cleavage of the C-N (C-S, in our case) bond.

### Product inhibition

**Fig 9:**
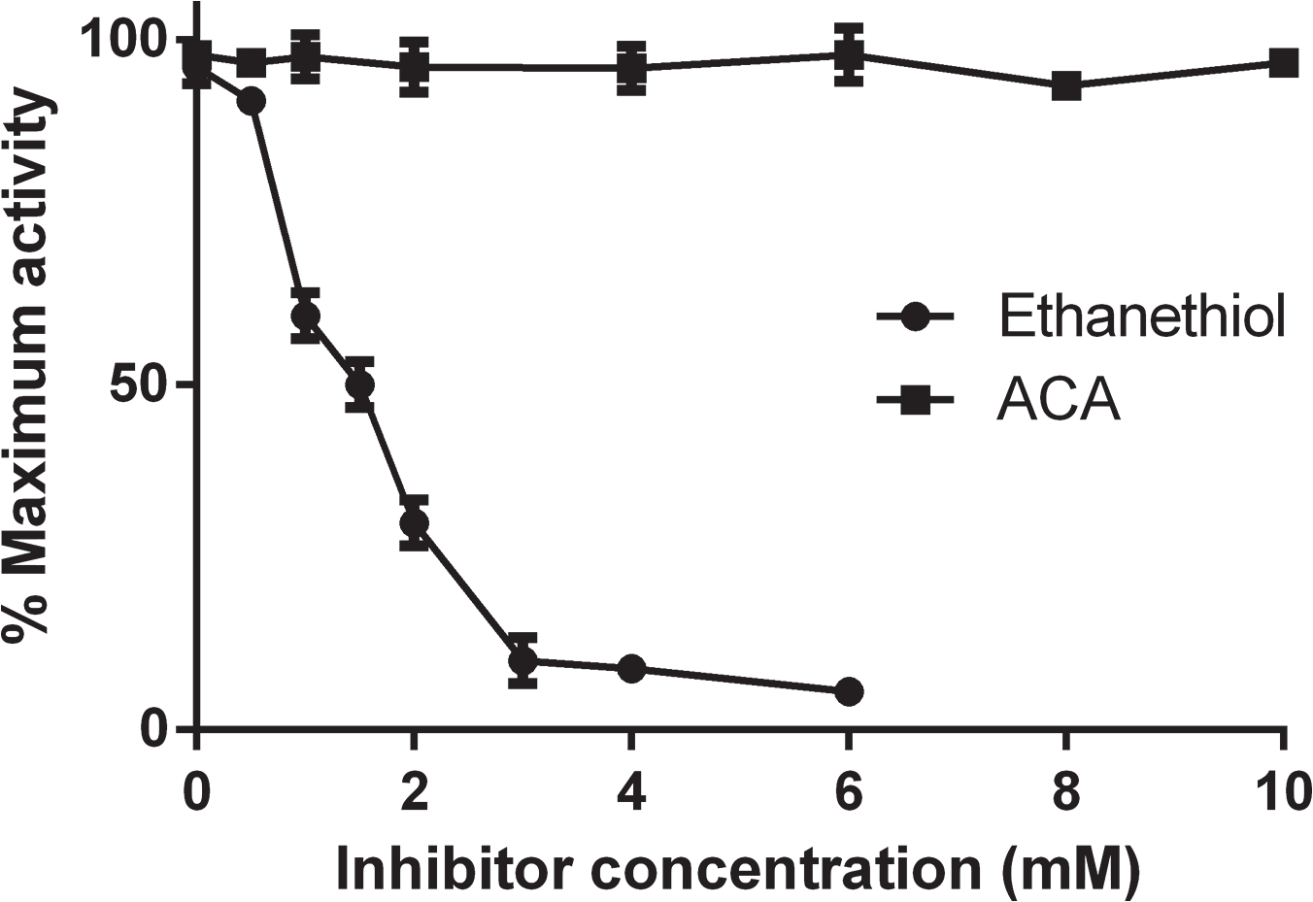
Effect of azepane-1-carboxilate (ACA) and ethanethiol on the activity of recombinant molinate hydrolase. Assays were performed with 0.5 mM molinate in 50 mM phosphate buffer at pH 7.4 at 30°C, with a protein concentration of 5 μg.ml^-1^ in the presence of different concentrations of ACA and ethanethiol. The data points represent the mean values from triplicate assays and the error bars are the corresponding standard deviations.

The effect of reaction products (azepane-1-carboxilate and ethanethiol) on molinate hydrolase activity was followed by an HPLC assay performed in the presence of different concentrations of those compounds. As shown in Fig 9, azepane-1-carboxylate does not have any effect, while ethanethiol inhibits molinate hydrolase. This inhibitory effect is likely to occur due to the interaction of the thiol group with the metal ion of the active site. This hypothesis was tested and confirmed by the observation that cysteine also inhibits the enzyme activity, while serine and methionine do not. In addition, other thiolated compounds, such as β-mercaptoethanol, also inhibited molinate hydrolase, suggesting that the coordenation of the thiol group with the catalytic metal renders the enzyme inactive. Thus, considering a mechanism of competitive enzymatic inhibition, the inhibition constant, K_i_, was determined to be 0.30 mM (GraphPad Prism version 6.00).

The decrease in enzyme activity when this product accumulates is in agreement with the behaviour of *G*. *molinativorax* ON4^T^. In previous studies it was demonstrated that this organism is not able to grow on molinate in axenic culture at molinate concentrations over 2 mM, due to the toxic effect of ethanethiol [3, 4]. At higher molinate concentrations, *G*. *molinativorax* ON4^T^ only grows in the presence of other consortium members, which are able to degrade ethanethiol, and thus are responsible for the removal of this sulphur compound [3, 4].

### Structure-guided engineering of molinate hydrolase

**Fig 10:**
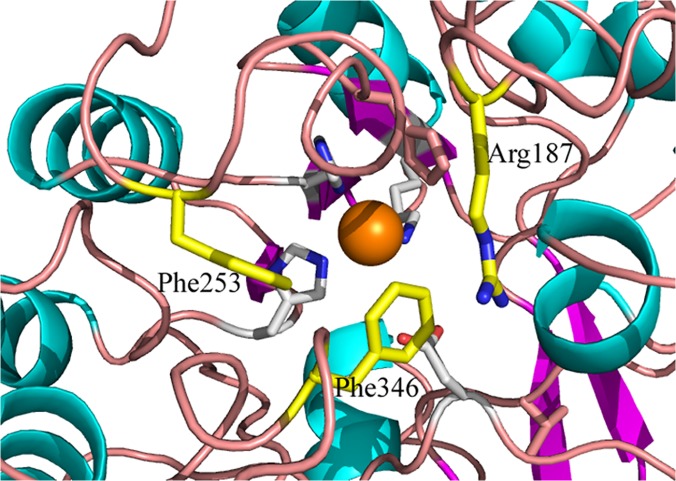
Site direct mutagenesis of molinate hydrolase. Detail view of the active site highlighting (in yellow) the residues that potentially restrict the access to the active site. In order to attempt to engineer the enzyme to degrade bulky thiocarbamates, single-site mutants of Arg187, Phe253 and Phe346 to alanine were produced.

Molinate hydrolase, like many other amidohydrolases, has the active site access restricted by the many loops that link the β-strands and α-helixes that surround the catalytic site. Some of these loops contain bulky residues that may act as gatekeepers. We selected Arg187, Phe253 and Phe346 as the most promising residues to play this role (Fig 10). Single point mutations to alanine of the three residues were produced.

**Fig 11:**
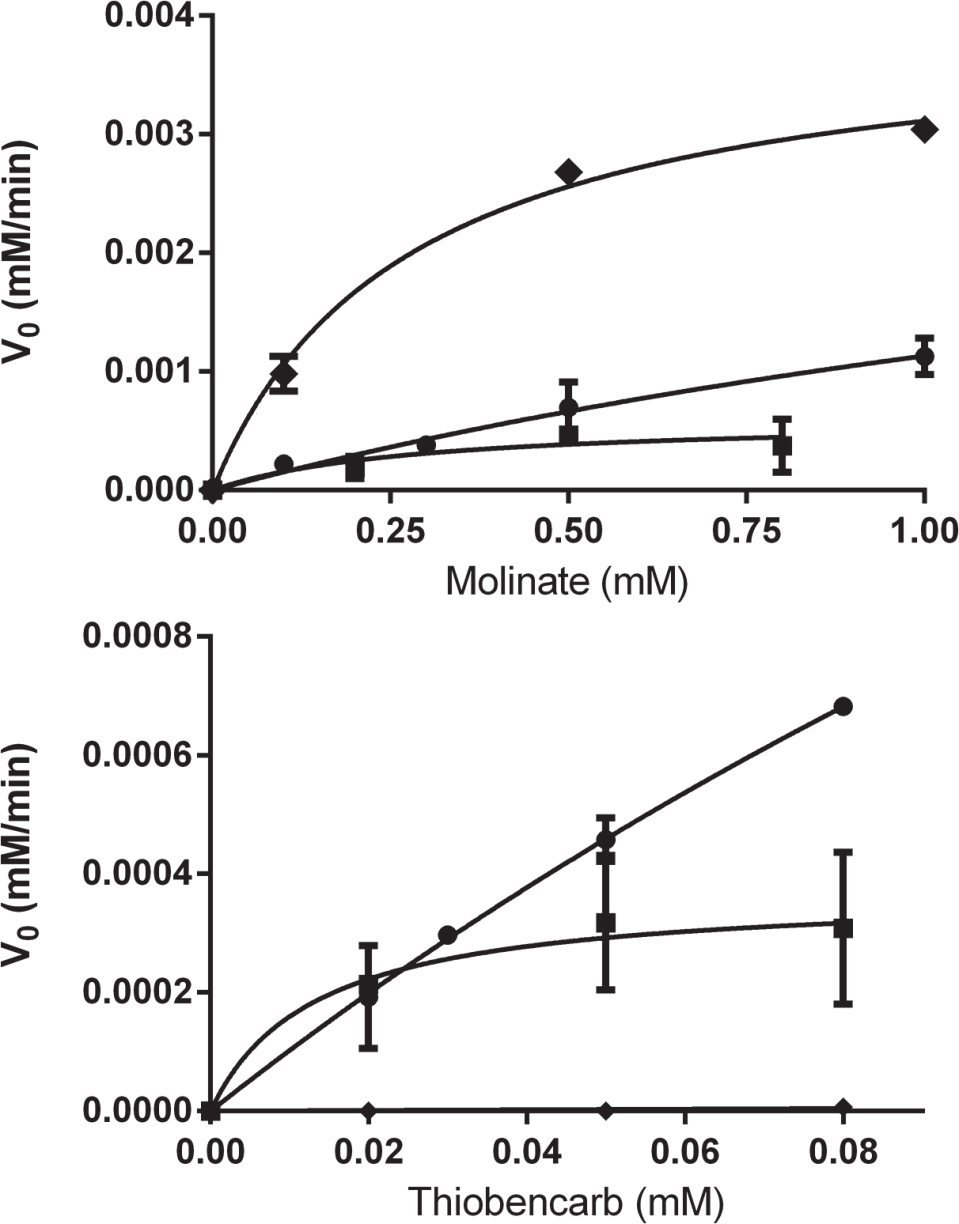
Molinate hydrolase activity kinetics. Dependence of the initial reaction rate on the substrate concentration (molinate, top; thiobencarb, bottom) by recombinant molinate hydrolase (♦), mutant Arg187Ala (●) and mutant Phe253Ala (■).

The activity of the aforementioned single mutants towards the degradation of molinate and thiobencarb was assessed at 25°C, pH 7.4 (Fig 11). Mutant Phe346Ala was unable to degrade molinate (data not shown). The other two mutants catalyzed the reaction although with distinct kinetics, Arg187Ala was significantly more efficient than Phe253Ala. When compared to the recombinant enzyme activity [5] and analyzed by the Michaelis-Menten model, Arg187Ala shows lower affinity towards molinate (K_m_ = 2.37 mM against K_m_ = 0.275 mM from the recombinant) while the turnover number remains very similar (Arg187Ala: k_cat_ = 38.1 min^-1^ and recombinant: k_cat_ = 39.7 min^-1^).

Thiobencarb is not processed by molinate hydrolase (Fig 11) as we have reported earlier [5]. By contrast, both mutants Arg187Ala and Phe253Ala degraded this thiocarbamate. Phe253Ala was consistently less efficient than Arg187Ala mutant, which may be due to a depletion of the catalytic metal associated with this specific mutation. Mutant Arg187Ala showed a k_cat_ / K_m_ ratio of 44.2 ± 6.5 mM^-1^min^-1^ which is in the same order of magnitude of the correspondent value obtained with molinate, an indication that Arg187Ala catalyses the reaction of both substrates (molinate and thiobencarb) with similar efficiency. Due to the low solubility of thiobencarb it was not possible to increase the substrate concentration to enable the accurate estimation of k_cat_.

We focused our study on thiobencarb because it is also applied in rice fields and is structurally diverse from molinate. Moreover, thiobencarb is a chlorinated thiocarbamate, a particular class that attracts high environmental concerns. Herein, we confirmed that the Arg187Ala mutant is able to degrade both thiocarbamates. Hence, both recombinant and Arg187Ala mutant enzymes are excellent prospective candidates for application in the bioremediation of rice field runoff water.

## Conclusions

In summary, we determined the crystal structure of molinate hydrolase, which allowed us to propose a catalytic mechanism for the reaction of molinate in light of the general structural and catalytic accumulated knowledge within the amidohydrolase superfamily. Furthermore, we disclosed why molinate excess in axenic *G*. *molinativorax* ON4^T^ culture inhibits its growth; molinate hydrolase is inhibited by ethanethiol, which is one of the products of molinate hydrolysis. The free thiol groups show propensity to occupy the free coordination sites of the catalytic metal.

The knowledge of the enzyme three-dimensional structure allowed us to design mutants able to degrade other thiocarbamates. One mutant was identified, Arg187Ala, that is able to catalyze the reaction with at least molinate and thiobencarb. The engineered specificity modulation seems to be accompanied by a decline of the affinity towards the original substrate, molinate. Nevertheless, mutant Arg187Ala can efficiently degrade structurally different thiocarbamates, thus paving the way for the development of a multi-herbicide decontamination system.

## Materials and Methods

### Production and purification of recombinant and mutant molinate hydrolase

To express the recombinant SeMet variant, the biomass of a freshly streaked plate from a fresh transformation of *E*. *coli* B834 (DE3) with expression vector pASK*molA* (obtained as previously described [5], was resuspended and grown for 2 h, at 37°C, 130 rpm, in 5 mL of LB medium containing 100 μg ml^-1^ ampicillin; appropriate amounts of the starter culture were used to inoculate the main culture (minimal media) in order to have an initial OD_600nm_ of 0.06.

For the expression of recombinant molinate hydrolase and mutant forms, *E*. *coli* JM109 or B834 (DE3) were transformed with expression vectors pASK*molA* and pASKmolAmt240/ pASKmolAmt282/ pASKmolAmt302/ pASKmolAmt187/ pASKmolAmt253/ pASKmolAmt346 (according to the mutated residue to alanine). The biomass from a freshly streaked plate was resuspended in 5 ml LB medium supplemented with 100 μg ml^-1^ ampicillin. Each resuspended plate was used to inoculate 1 L of main culture (LB with 100 μg ml^-1^ ampicillin).

Main culture, identical for all expressed forms of the protein, was grown at 30°C, 130 rpm until OD_600nm_ of 0.7; at that point, cells were subjected to cold shock and temperature was lowered to 15°C. Induction was performed at OD_600nm_ of 1.0 with 0.2 μg ml^-1^ anhydrotetracyclin. Cells were harvested (3985 x *g*, 20 minutes, 4°C, Avanti J26XPI, Beckman Coulter, rotor JLA 8.1000) after 18–20 h of incubation at 15°C with 130 rpm orbital shaking.

Purification protocol was also common to all expressed forms of molinate hydrolase. The cell pellet was resuspended in lysis buffer (10 ml per litre of culture; 150 mM NaCl, 100 mM Tris-HCl pH 8.0; 1 mM phenylmethanesulfonyl fluoride; 20 μg ml^-1^ DNase I; 250 μg ml^-1^ lysozyme) and stored at -20°C. Cells were disrupted by two passages through a FRENCH Press (Thermo Scientific). Unbroken cells and cell debris were removed by centrifugation at 34 895 x *g*, 4°C, 45 min (Avanti J26XPI, Beckman Coulter, rotor JA 25.50). Recombinant molinate hydrolase was purified from cleared lysate using a Strep-Tactin Sepharose gravity flow column (IBA) with a 2 ml column volume. Column was washed using Tris HCl 100 mM, 150 mM NaCl, pH8.0 and lysate loaded at 0.5 ml min^-1^; 1 ml fractions were eluted at 1 ml min^-1^ with Tris HCl 100 mM, 150 mM NaCl, pH 8.0, 2.5 mM d-desthiobiotin. Active fractions were pooled, concentrated (Vivaspin, 30,000 MWCO for L-SeMet protein and Amicon 10 000 MWCO for non- L-SeMet protein) and further purified by size exclusion chromatography using a Superose 12 10/300 GL column (GE Healthcare), connected to an ÄKTA Purifier 10 system with Unicorn 5.1 (GE Healthcare Bio-Sciences AB).

### Crystallization conditions

After purification, protein samples were concentrated up to 5–10 mg ml^-1^ (quantification performed by absorbance at 280 nm) and the initial buffer Tris HCl 100 mM pH 8.0, 150 mM NaCl was exchanged with Tris HCl 20 mM pH 8.0. The protein aliquots were centrifuged (13 000 rpm, 30 min at 4°C) prior to the crystallization trials which were carried out by both sitting-drop and hanging-drop vapour diffusion techniques at 20°C, using 24-well plate by mixing 1.5 μl of the protein solution with 1.5 μl of the reservoir condition and equilibrated against a 500 μL reservoir. Several commercial crystallization screening kits were tested. Crystals were obtained in one week using two different conditions: (1) 0.095 M sodium citrate tribasic dihydrate, pH 5.6–6.4, 19% v/v 2-propanol, 19% polyethylene glycol 4000, 5% v/v glycerol and (2) 0.1 M BIS-TRIS, pH 6.5, 45% polypropylene glycol P400. They could be directly flash frozen for data collection and it was observed that the better diffracting crystals were those grown using condition (1).

The initial X-ray diffraction studies with the SeMet derivative of recombinant molinate hydrolase allowed the structure determination of the metal-depleted form of the enzyme. Additional crystallization trials of the recombinant molinate hydrolase with crystallization condition (1), supplemented with either 4 mM or 8 mM zinc chloride were performed.

### Crystal structure determination of SeMet derivative of molinate hydrolase

Data collection and processing—A Single Wavelength Anomalous Dispersion (SAD) data set to 2.85 Å resolution was measured at the Selenium absorption K-edge from a flash cooled crystal on ESRF beamline ID14-4. The peak energy was selected from a fluorescence scan using CHOOCH [12]. Diffraction images were processed with the XDS Program Package [13] and the diffraction intensities converted to structure factors in the CCP4 format [14]. A random 5% sample of the reflection data was flagged for R-free calculations [15] during model building and refinement. A summary of the data collection statistics is presented in Table 1. The crystal belonged to one of the orthorhombic space groups *I*222 or *I*2_1_2_1_2_1_ with unit cell dimensions *a* = 128.44, *b* = 230.18, *c* = 264.83 Å. Matthews coefficient calculations [16] suggested the presence of 8 molecules in the asymmetric unit, with V_m_ = 2.24 Å^3^Da^-1^ and a predicted solvent content of 45%.

**Table 1:** Crystallographic data collection, processing and phase refinement statistics for the SeMet derivative.

***Data collection and processing statistics***	
Beamline	ESRF ID14-4
Detector	ADSC Q315r
Wavelength (Å)	0.9736
Data Processing	XDS/CCP4
Space Group	*I*2_1_2_1_2_1_
Unit cell parameters (Å,°)	
*a*	128.44
*b*	230.18
*C*	264.83
*α*, *β*, *γ*	90, 90, 90
Resolution (Å) ^a^	57.8–2.85 (2.95–2.85)
Nr. Observations	1343228 (134496)
Unique reflections	91555 (8907)
Multiplicity	14.7 (15.1)
Completeness (%)	100.0 (100.0)
R-merge (%) ^b^	11.2 (56.0)
<I/σ (I)>	21.3 (4.2)
R-pim (%) ^c^	3.2 (14.8)
Wilson B (Å^2^)	54.7
Z ^d^	4
Estimated V_M_ ^e^	4.48
Estimated Solvent Content (%)^e^	72.5
***Phase refinement statistics***	
Phasing power, anomalous	2.13
Anomalous R_cullis_	0.570
SHARP FOM, acentric	0.424
SHARP FOM, centric	0.106
SHARP FOM, overall	0.403
***Density modification statistics***	
Overall |E^2^| correlation ^f^	0.813
FOM after density modification ^f^	0.911

^a^ Values in parentheses refer to the highest resolution shell.

^b^ R-merge = merging R-factor, (Σ_hkl_ Σ_i_ |I_i_(hkl)—<I(hkl)>|) / (Σ_hkl_ Σ_i_ I(hkl)) × 100%

^c^ R-pim = precision independent R-factor, Σ_hkl_ [1/(N-1)]^1/2^ Σ_i_ |I_i_(hkl)—<I(hkl) >| / (Σ_hkl_ Σ_i_ I_i_(hkl)) × 100%[24]. For each unique Bragg reflection with indices (hkl), I_i_ is the i-th observation of its intensity and N its multiplicity.

^d^ Nr. molecules in the asymmetric unit.

^e^ According to Matthews coefficient [25].

^f^ from SHARP optimizing density modification procedure with SOLOMON [20].

Structure solution and crystallographic model building**—**The Se heavy atom substructure was determined with Shake-and-Bake [17] as implemented in the BnP interface [18], using the SAD data to 3.5 Å resolution. Based on the most likely asymmetric unit contents, 168 Se sites were expected, and the calculations were performed on both possible space groups. A few solutions (10 in 1000 trials) were obtained only in *I*2_1_2_1_2_1_ as indicated by a bimodal histogram of the minimal function (R_min_) for 1000 trial substructures. Since the refined heavy atom occupancies did not show a sharp drop near the expected number of sites, the top 100 sites were input to a maximum-likelihood heavy-atom parameter refinement using autoSHARP [19]. The autoSHARP calculations eliminated some sites and located others, the final total number being 81. The centroid SHARP phases were further improved by an optimizing density modification procedure using SOLOMON [20], which suggested a solvent content of 63.8%. These results prompted a reinterpretation of the asymmetric unit contents, and it was concluded that there were only 4 monomers instead of 8 as initially expected, corresponding to V_m_ = 4.48 Å^3^Da^-1^ and a predicted solvent content of 72.5%. Phasing and phase refinement statistics are listed in Table 1. The electron density map obtained was of excellent quality and about 1824 residues of the expected 1984 could be built and sequenced automatically with Buccaneer/REFMAC [21, 22]. The autobuilding procedure gave values of R and R-free of 0.252 and 0.275, and the model was completed with Coot [23].

### Data collection, processing and refinement of the zinc-bound molinate hydrolase

Crystals from the zinc-bound molinate hydrolase were either isomorphous to the orthorhombic SeMet recombinant molinate hydrolase crystals or belonged to the monoclinic space group *C*2. The position of the bound zinc ions was determined from a single wavelength anomalous diffraction data collection to 2.27 Å resolution at the Zn absorption edge, determined experimentally on ESRF beamline ID23-1 (λ = 1.265 Å; Grenoble, France). The diffraction images were processed similarly to the SeMet derivative dataset. The crystal belonged to space group *C*2, with cell dimensions *a* = 367.48, *b* = 98.86, *c* = 131.20 Å and *β* = 109.59°. Initial molecular replacement phases were generated with PhaserMR [26], using as initial model one monomer of the SeMet derivative structure; in total, 8 monomers were present in the asymmetric unit. The top 8 positive peaks in the F^+^—F^-^ anomalous difference Fourier map were assigned to the Zn^2+^ ions; it was observed that they were located in equivalent positions in each monomer. Despite the modest peak height, between 5 and 7σ, its position was consistent with metal coordination geometry and with the position of the metal ions in structure-related amidohydrolases. Furthermore, site direct mutagenesis of the metal coordinating residues confirmed a decline in enzymatic activity. The occupation of the metal ions was set to 0.4 during refinement to adjust their isotropic atomic displacement parameters to their local environment. Data collection and refinement statistics are shown in table 2. The final model was obtained after further cycles of building and refinement, carried out with Coot [27] and REFMAC5 [28], respectively. Non-crystallographic symmetry was taken into account during refinement. Figures of protein structures were generated using PyMol [29]. Atomic coordinates have been deposited in the Protein Data Bank with an accession code: 4UB9.

**Table 2:** Data collection and refinement statistics.

*Crystal data and data-collection statistics*	*zinc-bound molinate hydrolase*
Wavelength (Å)	1.265
Space Group	*C* 2
Unit-cell parameters (Å)	
*a*	367.6
*b*	99.0
*c*	131.3
β	109.6
Resolution range (Å)	100.0–2.27 (2.35–2.27)
Observed reflections	545964 (31520)
No. of unique reflections	
Completeness (%)	93.6 (66.3)
Multiplicity	2.66 (1.56)
<I/σ(I)>	12.2 (1.73)
R-merge (%) ^a^	5.8 (47.8)
***Refinement***	
R_cryst_(%)	20.1
R_free_(%)	22.9
RMSD for bonds (Å)	0.0131
RMSD for angles (°)	1.5587
RMSD for chiral volumes (Å^3^)	0.0911
Average main chain B-factor (Å^2^)	39.17
Average side chain B-factor (Å^2^)	52.67
Average zinc B-factor (Å^2^)	56.35
Average water B-factor (Å^2^)	39.71
Ramachandran plot statistics (%)	
Favoured regions	96.16
Allowed regions	3.84

^a^ R-merge = merging R-factor, (Σ_hkl_ Σ_i_ |I_i_(hkl)—<I(hkl)>|) / (Σ_hkl_ Σ_i_ I(hkl)) × 100%

Values in parentheses are for the highest resolution shell.

### *In vitro* mutagenesis

To confirm the metal-dependent active site and for the structure-guided engineering of molinate hydrolase, site-directed mutagenesis with Pfu DNA polymerase (Thermo Scientific) or PfuTurbo DNA polymerase (Agilent Technologies) was performed. Mutagenic primer sequences (forward and reverse; sequence provided upon request) were designed with QuickChange Primer Design Program (Agilent Technologies) and purchased from Sigma-Aldrich. Each residue (Lys240, His282 and His302 for the metal-dependent active site; Arg187, Phe253 and Phe346 for the structure-guided engineering of molinate hydrolase) were singly switched to alanine. PCR was carried out based on the manufacturer’s recommendations (Thermo Scientific/Agilent Technologies). PCR products were checked on 1% agarose/ethidium bromide DNA gel and correct sized samples (around 4700 bp) were treated for 3 h at 37°C with DpnI restriction enzyme to achieve pASKmolA template degradation, following transformation to *E*. *coli* DH5α. Selected colonies were picked and grown overnight at 37°C, 150 rpm in 10 ml LB supplemented with 100 μg ml^-1^ ampicillin. Mutant plasmid DNA aliquots were prepared using GRS Plasmid Purification Kit (Grisp Research Solutions) and the desired mutations were confirmed by sequencing (STABvida, Portugal).

Mutant proteins correct folding was evaluated by circular dichroism (CD). CD spectra were recorded by a J-815 (Jasco, Japan) spectrometer, equipped with a Peltier temperature control system, set at 20°C. Protein samples at 0.1 mg ml-1, diluted in TrisHCl 10 mM pH 8.0. Spectra recorded in a 1 mm pathlenght quartz cell (Hellma Analytics, Germany) from 260 to 190 nm, at 50 nm min-1, D.I.T. of 2s, data-pitch 0.2 nm and 16 accumulations per measurement. Spectra smoothed using the Savitzky–Golay algorithm and corrected for the blank sample.

### Enzyme activity assays and evaluation of inhibitors

Recombinant and active-site mutant molinate hydrolase activity was assayed by following substrate depletion through high performance liquid chromatography (HPLC), as previously described [4]. Assays were carried out using 0.1 μM recombinant molinate hydrolase or active-site mutants and 0.5 mM molinate, in 50 mM phosphate buffer pH 7.4 Molinate depletion was followed for up to 30 min at room temperature (~25°C). The initial velocity of molinate degradation was calculated from linear regression of the substrate concentration versus time plot.

The effect of ACA and ethanethiol on molinate hydrolase activity, in concentrations up to 10 and 6 mM, respectively, was studied by adding these reaction products to the activity assays. ACA was obtained as previously described [3]. The inhibition constant, Ki, was determined using the competitive enzyme inhibition algorithm embedded in GraphPad Prism version 6.00. The effect of the thiol group on molinate hydrolase activity was further confirmed by UV spectrophotometry. Control reaction was performed by incubating 1 μM of recombinant molinate hydrolase with 0.5 mM molinate and substrate depletion was followed at 220 nm (molinate maximum absorption wavelength). Then, 2-mercaptoethanol (3 mM), L-cysteine (1.5 mM), L-serine (1.5 mM) and L-methionine (1.5 mM) were added individually, to further confirm the effect of the thiol group on enzyme inhibition.

Enzymatic kinetic assays for mutants Arg187Ala, Phe253Ala and Phe346Ala were performed by HPLC. Protein concentration was fixed at 0.1 μM, while substrate concentrations were varied (molinate, 0.1 to 1 mM; thiobencarb 0.02 to 0.08 mM). As before, the initial velocity of molinate degradation was calculated from linear regression of the substrate concentration versus time plot. Then, kinetic parameters were calculated using non-linear regression of initial speed values for increasing substrate concentrations and a E_t_ value of 0.1 μM, using GraphPad Prism version 6.00.
